# Traditional knowledge, uses, and perceptions of mushrooms among the Wixaritari and mestizos of Villa Guerrero, Jalisco, Mexico

**DOI:** 10.1186/s43008-019-0014-6

**Published:** 2019-09-16

**Authors:** Mara Ximena Haro-Luna, Felipe Ruan-Soto, Laura Guzmán-Dávalos

**Affiliations:** 1 Maestría en Ciencias en Biosistemática y Manejo de Recursos Naturales, Universidad de Guadalajara, Zapopan, Mexico; 2 Departamento de Botánica y Zoología, Universidad de Guadalajara, Apdo. postal 1–139, 45101 Zapopan, Jalisco Mexico; 3 Instituto de Ciencias Biológicas, Universidad de Ciencias y Artes de Chiapas, 29039 Tuxtla Gutiérrez, Chiapas Mexico

**Keywords:** Cosmovision, Ethnomycology, Huichol, Medicinal mushrooms, Mycophilic, Wild edible fungi

## Abstract

The relationship between humans and nature is defined by culture. Accordingly, the use, conceptions, and perceptions of resources differ among cultural groups, even among those inhabiting the same region or those who come into contact with the same biota. In particular, mushrooms evoke a wide range of sentiments. During ethnobiological tours in Mexico, semi-structured interviews were carried out with 37 individuals of each community, from ten Wixarika and mestizo communities, living in the same locality and sharing similar resources, in the municipality of Villa Guerrero in northern Jalisco, Mexico. Furthermore, informal interviews with four Wixarika and five mestizo key informants were conducted. The topics treated were regarding the traditional nomenclature and classification, uses, and knowledge of mushrooms and related practices. Wixarika names of 37 mushroom species with edible, medicinal, and recreational uses were recorded. In addition, the Wixaritari were found to associate toxic mushrooms with the divine, as evidenced by one case of the use of mushrooms as a hierophanic agent. Each culture’s knowledge of the phenology and ecology of mushrooms was recorded in addition to data highlighting the cultural exchange between the Wixaritari and mestizos. However, a loss in the knowledge and practices concerning mushrooms was observed as a result of social changes. Even so, both cultures prefer mushrooms to other foods, including meat, especially *Volvariella bombycina* and *Pleurotus djamor*.

## INTRODUCTION

Environmental, biological, and cultural factors have influenced the relationships established between humans and the different components of nature. As a result of human-nature interactions, societies have constructed different sets of knowledge over time. Traditional ecological knowledge represents the culmination of wisdom, practices, and beliefs transmitted generationally regarding the relationship of living things (Berkes et al. 2000). The knowledge sets of human groups are not dissociated but rather form part of a worldview that unites the past with the future and integrates visible elements and materials with those that are subjective and mystic as well as everyday practices (Escobar-Berón 2002). The perception that humans form part of a greater whole is mainly observed in indigenous groups, contrasting with the anthropocentric Western vision introduced during colonization in which humans do not form part of nature but instead utilize nature to obtain resources (Plumwood 2006).

Mexico is a culturally and biologically diverse country where the traditional knowledge of each region and among its peoples has been conserved to a certain extent (Boege 2008). The use of mushrooms has been documented in 15 of the 68 indigenous groups of Mexico and in various mestizo communities living in rural areas (Garibay-Orijel and Ruan-Soto 2014) —understanding Mexican mestizos as those resulted by the mixture of Amerindian and European ancestors (Silva-Zolezzi et al. 2009). Each culture has been related in different ways to fungi, given way to a great plurality of ethnomycological knowledge (Ruan-Soto et al. 2014), according to the identity of each person and the place where they develop (Mapes et al. 2002). Throughout the country, there are sites where indigenous and mestizos groups co-exist. One example is in the northern region of Jalisco where both Wixarika (or Huichol) and mestizo groups are found. The Wixaritari (sing. Wixarika) are known for their culture and the way to interpret the world (Neurath 2013). In contrast, the mestizos of this region have only a Hispanic cultural identity and aim to maintain a certain distance with the indigenous world that is considered inferior (Barragán-López 1990).

The Wixarika and mestizo cultures in the northern region of Jalisco are isolated, mainly by the rugged topography, and this has made them dependent upon natural resources for their subsistence. They have different relationships with nature and different concepts, perceptions, forms of use, and management of biological resources (Neurath 2000). In this study, we therefore considered aspects related to the use, management, and perceptions of mushrooms and related cultural practices in these cultures residing in the same or different communities in northern Jalisco, Mexico.

## METHODS

### Study area

Villa Guerrero, located in the northern zone of Jalisco, Mexico (21°51′–22°11′ N, 103°52′ W), has 47.7% of the surface corresponding to mountainous terrain with slopes greater than 15° (IIEG 2018), with altitude ranges from 980 to 2360 m a.s.l. (INEGI 2019). It has a semi-warm, semi-humid climate with an annual average temperature of 18.3 °C and an average annual rainfall of 700 mm. These conditions result in different types of vegetation: pine-oak and oak forests dominant in the higher zones, subtropical scrubland and grasslands in the lower zones, and different successional states as a result of human activity (CONABIO 2012; IIEG 2018; INEGI 2019).

In the municipality, there are 62 villages. Of these, eight had only two houses and 21 had a single house; according to the 2019 Population and Housing Census (INEGI 2019), the total population was 5938. Amongst these, 6.04% were Wixaritari residing in indigenous communities and some in towns together with mestizos. Of the total population, 64.6% experienced multidimensional poverty, and only 13.6% had access to adequate nutrition and 11.8% to health services (IIEG 2018). The main economic activities were commerce, agriculture, and livestock ranching (Shadow 2002; IIEG 2018). A low degree of connectivity through roads and highways is present (INEGI 2019).

### Data collection and analysis

**Fig. 1:**
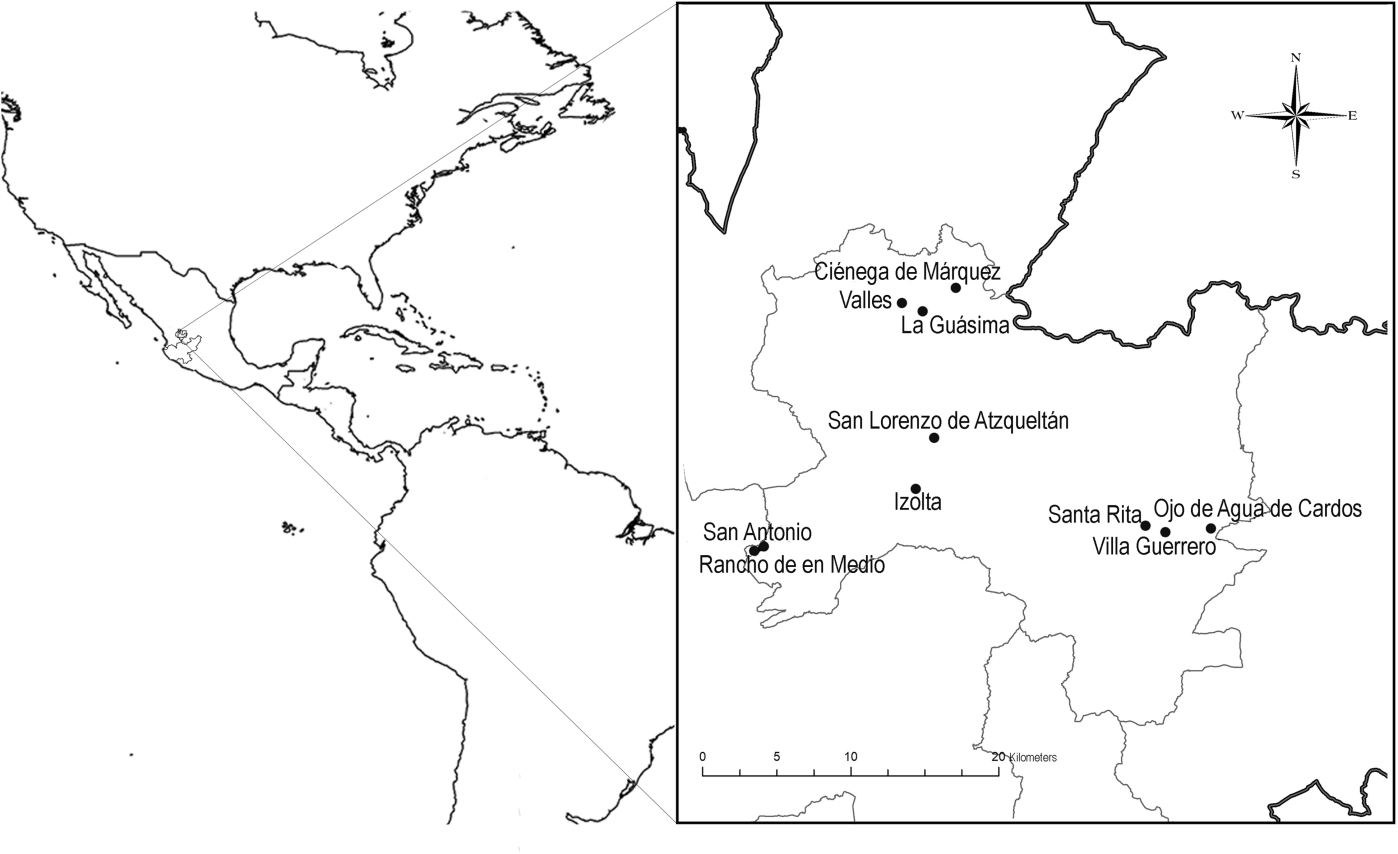
Map showing the communities where interviews were performed and mushrooms were collected

The data were obtained during fieldwork from February 2016 to March 2018 in five mestizo communities (Ciénega de Márquez, Izolta, La Guásima, Ojo de Agua de Cardos, and Santa Rita), three Wixarika communities (Manillas or Rancho de En Medio, San Antonio, and Los Valles), and two communities where both cultural groups have cohabitated for approximately 70 years (San Lorenzo de Atzqueltán and Villa Guerrero) (Fig. 1). In the first phase of the fieldwork, permission was requested from the municipal and communal authorities and from each person interviewed. The objective and intention of the research study was explained following the ethical code of the Latin American Society of Ethnobiology (SOLAE, Spanish acronym; Cano-Contreras et al. 2015).

Ethnobiological excursions (walks-in-the-woods) were carried out in the rainy season, from June to September of 2016 and 2017, in the company of key informants in or near their lands or community; in pine, oak, or pine-oak forests, subtropical scrubland, and grasslands. During these excursions, local mushroom species named or recognized by the interviewees were considered as ethnotaxa, which could include one or several taxonomically recognized species (Zent and Zent 2011). The mushrooms were photographed in the field to create a visual stimulus and to confirm the correspondence of each species with their traditional name (Montoya et al. 2012). The mushrooms were described in fresh condition, dehydrated, and determined using conventional mycological techniques based on their macroscopic and microscopic characteristics (Largent et al. 1977; Largent 1981), and specialized literature. The mushrooms were then deposited in the Mycological Collection of the Dra. Luz María Villarreal de Puga Herbarium of the Botanical Institute at University of Guadalajara (IBUG).

Semi-structured interviews (Campbell et al. 2013) and informal interviews (Moeller et al. 1980) were carried out with 16 Wixarika women, 17 mestizo women, four Wixarika men, and eight mestizo men aged 30 to 84 years old. The interviewees were selected based on the snowball method described by Noy (2008), i.e., people recognized by those in their communities as knowing most about mushrooms. During the interviews, themes explored related to the knowledge of wild mushrooms, including classification, naming, ecology, phenology, collection, culinary aspects, and origin myths of mushrooms, as well as other topics brought to our attention.

The interviews were recorded in a field diary and, when authorized, also as an audio file. The recordings were subsequently transcribed and systematized according to theme. A categorical analysis was performed following Echeverría (2005), in which the results and observed patterns were described per designated category.

## RESULTS AND DISCUSSION

### Nomenclature and traditional classification

Thirty-seven species were recognized between both cultural groups. Of these, 36 were known by the Wixaritari, which belonged to 33 ethnotaxa (Table 1). The Wixaritari considered 20 ethnotaxa to be edible, four without use, eight toxic, one medicinal, and *Ganoderma oerstedii* both as food and medicine. Mestizos recognized 13 ethnotaxa that included at least 14 species (Table 1), eleven were considered edible, one with no use, and one (three species) toxic. Four of the ethnotaxa had two or more common names. The Wixaritari and the mestizos considered mushrooms to be a distinct group from plants and animals. As in ethnic groups in Mexico (Mapes et al. 1981; Elizondo 1991; Lampman 2007; Ruan-Soto et al. 2007; Hunn et al. 2015) and other parts of the world such as Indonesia (Ellen 2008) and the Brazilian Amazon (Cardoso et al. 2010), the Wixaritari and the mestizos were able to identify the ecological, phenological, and morphological characteristics of the mushrooms. The criteria used by both groups to distinguish mushrooms from plants related to their growth habits and life-cycles and were similar to those used by the Hoti of the Venezuelan Amazon (Zent et al. 2004). These three groups argue that mushrooms do not have leaves or flowers, can grow from dead trees, and only appear for a short time once a year.

**Table 1:** Species of mushrooms, vouchers, uses, and traditional names recognized by the Wixaritari and mestizos

Species			Traditional name	
	Specimens^a^	Local use	Wixarika	Mestizo
*Agaricus campestris*	234	Edible	*Pɨsɨ, Pixɨxɨitsi*	Hongos de tierra, Sombrillita
*Amanita basii*	291	Edible	*Yekwá*	Hongo real, Hongo de la sierra
*Amanita laurae*	258	Edible	*Yekwá*	Hongo real, Hongo de la sierra
*Amanita muscaria*	283	Toxic	*Yekwá ‘itaikarieya*, *Yekwá kutsiyari*	–
*Amanita* sect. *Vaginata*	14, 297	Edible	*Huukú yekwá*	–
*Armillaria mellea* group	391	Edible	*Aruxi*	–
*Bolbitius* spp.	81	Toxic	*–*	Pasojito de burro
*Rubroboletus dupainii*	357	Edible	*Nema xure*	–
*Boletus edulis* group	265	Toxic	*Nema ‘itaikarieya*, *Nema kutsiyari*	–
	133	Edible	*Maxa nema*	–
*Calvatia cyathiformis*	229	Edible	*Temole*, *Tɨxi*	Bolitas de llano
*Calvatia* sp.	232	Edible	*Tapunaxe*, *Temole*, *Tɨxi*	Chapeteadas, Bolitas rojas
*Cantharellus cibarius*	23	Edible	*Tuutuxi*	Flores
*Ganoderma oerstedii*	390	Medicinal/ Edible	*Tuaxá naká*	Oreja de pino
*Gymnopus* sp.	257	Edible	*Wakanari*	–
*Hypomyces lactifluorum*	354	Edible	*Nakare*	–
*Lactarius* aff. *waltersii*	360	Toxic	*Tsurakaixi ‘itaikarieya*, *Tsurakaixi kutsiyari*	–
*Lactarius indigo*	167, 348	Without use	*Tsurakaixi ‘aikutsi*	–
*Lentinus levis*	256	Edible	*Huukú naká*	–
*Lyophyllum* spp.	25	Edible	*Atsi xɨté-xi*	–
*Marasmius oreades*	241	Edible	*Wakanari*	Corralitos
*Panaeolus antillarum*	242	Toxic	*Yekwá yɨyɨwi*	Pasojo de burro
*Pleurotus opuntiae*	104	Edible	*Naká nakari*	Oreja de nopal
*Psilocybe cubensis*	240	Toxic	*Yekwá yɨyɨwi*	Pasojito, Hongo de raja
*Pycnoporus sanguineus*	116	Medicinal	*Naká mɨxuxure*	–
*Ramaria* sect. *Botrytis*	7, 140	Toxic	*‘Ixuriki ‘itaikarieya*, *‘Ixuriki kutsiyari*	–
*Ramaria fennica*	12	Without use	*‘Ixuriki kuamoyé*	–
*Ramaria* sp. 1	138	Edible	*Ixuriki*	–
*Ramaria* sp. 2	141	Without use	*‘Iwi ‘ixuriki*	–
*Ramaria* sp. 3	142	Without use	*‘Ixurikiri mɨtataxawi*	–
*Russula* spp.	232	Edible	*Tsurakaixi*	–
*Schizophyllum commune*	107	Toxic	*Naká ‘itaikarieya*, *Naká kutsiyari*	–
*Suillus* spp.	275	Toxic	*Nema ‘itaikarieya*, *Nema kutsiyari*	–
*Ustilago maydis*	113	Edible	*Ki’au*	Cuervos, Cuitlacoche, Huilancoches, Pitacoche
*Volvariella bombycina*	244	Edible	*‘Utuxa yekwá*	Hongo de ochote

^**a**^Collector M. Haro-Luna

The Wixaritari called mushrooms *yekwá* in their language, whereas mestizos used the Spanish term *hongos* (mushrooms). Both cultural groups named specific mushrooms based on their ecological and morphological characteristics. The mestizos used simple or compound names that were usually descriptive (Berlin 1992). For example, *chapeteadas*, meaning rosy cheeks, was referred to the reddish colour in *Calvatia* sp., or *corralitos* (small corral) after the growth of *Marasmius oreades* in fairy rings. On the other hand, the compound names often began with the shape of the mushrooms, such as *oreja* (ear) or *hongo* (typical mushroom with cap and stipe), followed by the place or tree where the species grows, for instance, *Volvariella bombycina* is called *hongo de ochote* (mushroom from *ochote, Ipomea intrapilosa* wood).

*Ustilago maydis* was the species with most common names; even so, it was previously thought that this fungus was not traditionally consumed in Jalisco (Guzmán-Dávalos 1992). The mestizos called this *cuervo* (raven) for the black colour and its relationship with maize. In addition, the names *huilanconche* and *pitacoche* were recorded. These latter two terms are phonetic modifications of the term *cuitlacoche* (corn smut), a term which originated in central Mexico (Guzmán 2008).

Meanwhile, the Wixaritari named mushrooms with a single word, such as *yekwá* (*Amanita laurae* or *A. basii*) or *wakanari* (*Marasmius oreades*), or with two words, such as *utuxa yekwá* (*Volvariella bombycina*), *utuxa* being their name for *Ipomea intrapilosa*. When a binomial name was used, the first word was related to the plant or animal association, and the second to the form, although the order was reversed in some cases, with the first word referring to morphology and the second to colour. The names of mushrooms considered toxic were accompanied by the epithet ‘*itaikarieya*, from ‘*itaikari*, which means essence, spirit, or ghost, such as *yekwá* ‘*itaikarieya* (*A. muscaria*) or *kutsiyari* after *Kutsi*, who was the grandmother creator and first woman in Wixarika mythology, who germinated all plants and living creatures of the world (Perrin 1994; Neurath 2002; Iturrioz-Leza 2004). These concepts lack a literal translation, thus the Wixaritari have referred to these mushrooms in Spanish as *hongos de Dios* (mushrooms from God).

Some mushroom names used by mestizos were introduced by the Wixaritari. *Cantharellus cibarius* was called *flores* (flowers), which is the literal translation of the Wixarika name, *tuutuxi*. This name and the use of this mushroom spread from the Wixaritari to the mestizos during the government campaign to eradicate malaria in the 1960s (Cervantes-González 1978), during which trained mestizos worked for many years in Wixarika communities. When selling *Amanita basii* or *A. laurae*, the Wixaritari translated the name *yekwá* to *hongo real* (true mushroom) in Spanish. In this case, *real* refers to “reality” because it is an edible mushroom within the tangible realm of the cosmos. In contrast, *A. muscaria* or *yekwá kutsiyari* forms part of the spiritual realm and should not be eaten.

### Ecology

In the municipality, mushrooms were associated with the maintenance of life and natural cycles. Individuals also mentioned the capacity of mushrooms to degrade organic matter and recognized that conditions of humidity and shade were necessary for mushrooms to be produced, similar to the knowledge of other communities in central Mexico and Colombia (Vasco-Palacios et al. 2008; Jasso-Arriaga et al. 2016). Mushrooms were considered to be a source of food for humans and animals, although some individuals warned that animals eat a wide variety of mushrooms that may or may not be edible for humans.

Additionally, both groups understand that distinct mushrooms species are found in different habitats. The mestizos had greater contact with areas of subtropical and disturbed scrubland, and they were able to recognize the different sites where species grow and their phenology. Meanwhile, the semi-nomadic behaviour of the Wixaritari (Neurath 2000) had favoured their knowledge of a wide variety of species and their distribution in the different ecosystems of the municipality. The Wixaritari considered that some mushrooms were limited to areas with particular vegetation type because of the connection between mushrooms and trees. For this reason, they disapproved of the burning of vegetation performed by mestizos because this practice affected the production of mushrooms and other resources necessary for their subsistence. Although both groups carried out this activity, mestizos perform burning over more extensive areas of land so it could be used for grazing or harvesting food for livestock (Torres 2000). In contrast, the indigenous populations practiced burning, according to their population density, in small areas without woody vegetation (Barragán-López and Linck 1994).

Individuals only collected one or few fungus species at a time. Collecting trips were planned based on knowledge on the phenology of the species. For example, *Agaricus campestris*, *Calvatia cyathiformis*, and *Marasmius oreades* mushrooms first appeared in the last week of June, after the first rains. During the first half of July, the Wixaritari observed the emergence of *Amanita muscaria*, and knew that *A. basii* and *A. laurae* would be found several weeks later. The mestizos waited until the final days of July to search for *Pleurotus djamor* and *Volvariella bombycina* in ravines or along steep slopes. Meanwhile, the Wixaritari preferred to search for these latter species at the end of August and beginning of September. In August, they also went to oak forests to search for *Boletales*. *Ustilago maydis* was looked for between July and August. Only *Amanita* sect. *Vaginata* species and *Lentinus levis* were collected opportunistically. During September, the mestizos continued to consume *P. djamor*, whereas the Wixaritari preferred *Cantharellus cibarius*. Both groups considered coprophilous species to be present throughout the entire rainy season.

**Fig. 2:**
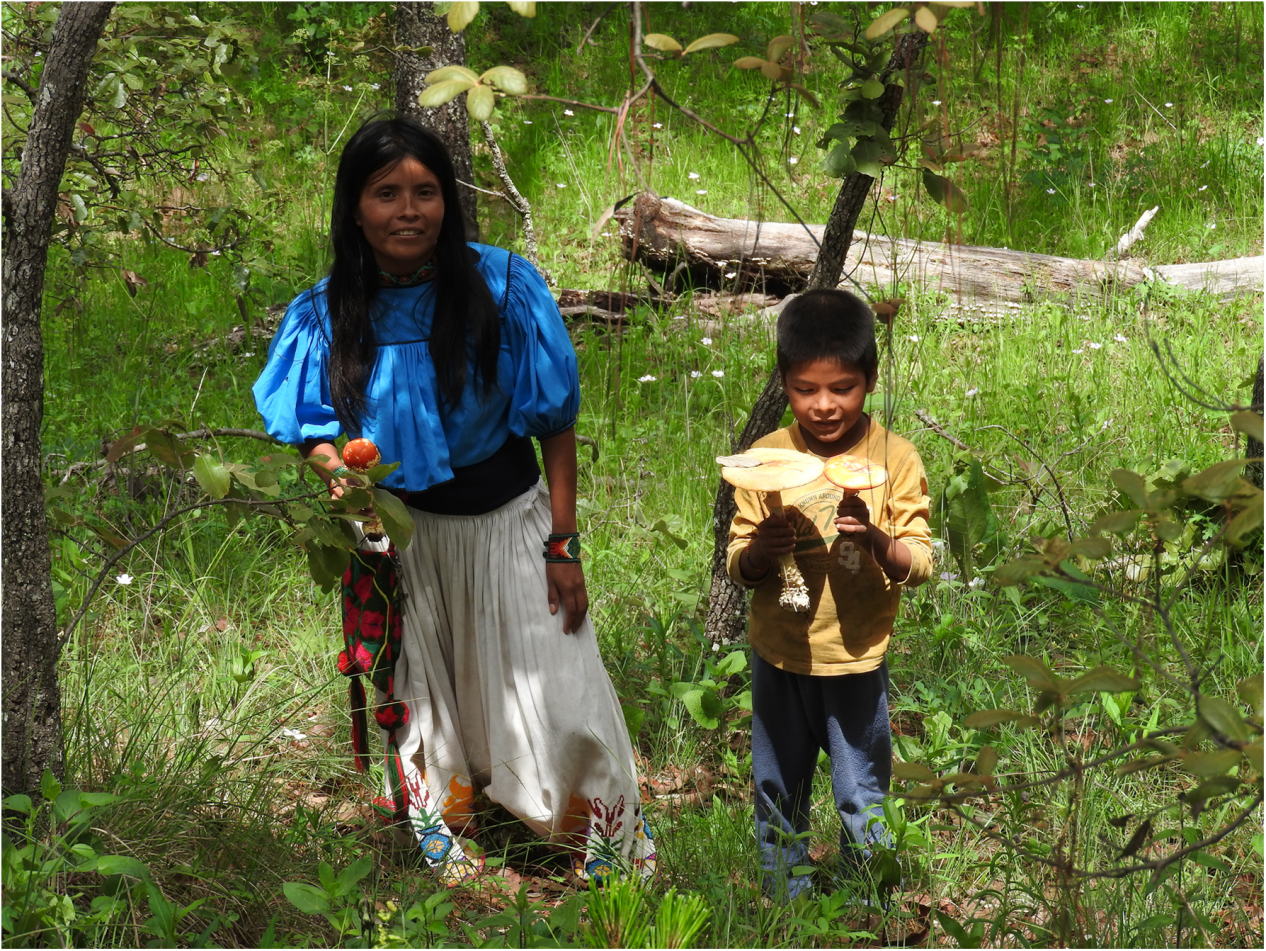
Robertina Valdez, Wixarika woman teaching her son how to recognize mushrooms

In the centre of Mexico, women are usually in charge of collecting and selling mushrooms or, when both genders carry out this practice, men traverse greater distances because women often carried or took children with them and thus were slower (Montoya et al. 2003, 2008). However, in the studied communities, the mushroom collection was carried out by both women and men, often accompanied by their children making it a family activity (Fig. 2).

In contrast to the state of Hidalgo and Valle de México, where the people consider that mushrooms develop and emerge at night-time because they are related with the lunar phases (Mariaca et al. 2001; Moreno-Fuentes and Bautista-Nava 2006), the Wixaritari and mestizos attributed the emergence of mushrooms to humidity rather than the time of day. Nevertheless, mestizos considered that mushrooms emerge at sunrise following nocturnal rains. In this regard, the Wixaritari made an anthropomorphic analogy. For them, mushrooms, comparable to humans, can be born during the day or night. Similar personifications of mushrooms have been reported in other ethnic groups.

**Fig. 3:**
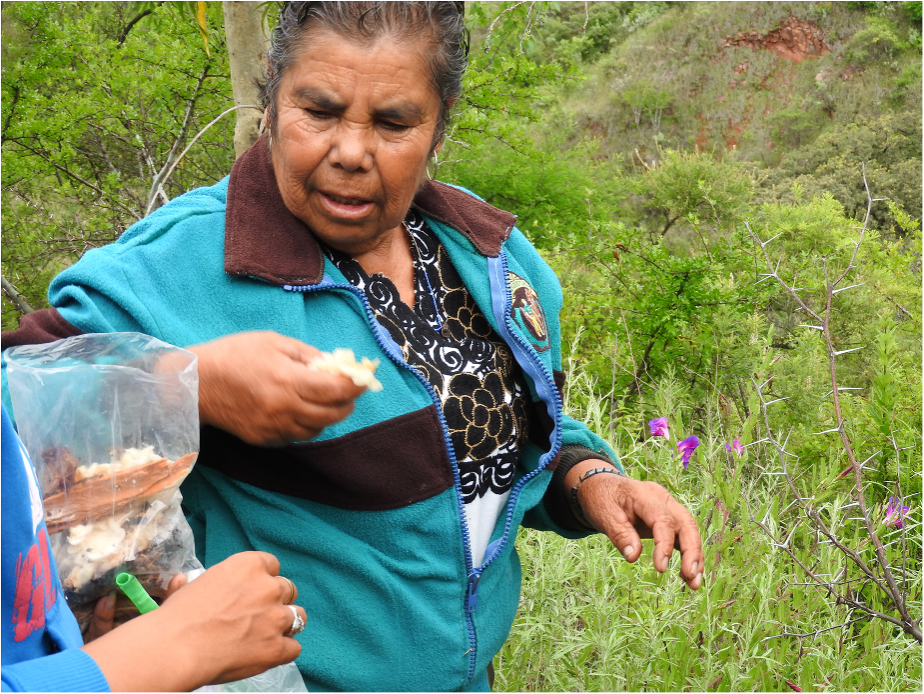
Doña Caritina, mestizo woman recollecting orejas de ochote (*Pleurotus djamor*) with her daughter

Mestizos and few Wixaritari cut fallen branches and trunks of the *ochote* (*Ipomea intrapilosa*) tree during winter and early spring to allow the wood to dry. Then by the beginning of the rainy season, *Pleurotus djamor* and *Volvariella bombycina* could begin to grow (Fig. 3). This practice is similar to that performed in the municipalities of Tlanchinol and Huehuetla in Hidalgo (Moreno-Fuentes and Bautista-Nava 2006). Trunks were hidden between weeds or underneath trees in strategic places to search for mushrooms, upon return between the months of August and September. However, because of the uncertainty related to the emergence of mushrooms, a series of beliefs had risen in this regard. For example, it was thought that only female trees could generate mushrooms, similar to how only women may have children.

### Uses

#### Edible mushrooms

The Wixaritari recognized 21 species of edible mushrooms, and the mestizos identified 11 species. Both groups preferred certain species, but in general mushrooms were valued as a food source and more appreciated than meat. It was unusual to encounter individuals who did not like their flavour. According to Ruan-Soto et al. (2013), these are indicators of mycophilia. Linking mushrooms with meat or the perception that mushrooms are better than meat is common in Mexico (Garibay-Orijel et al. 2007), and in some other countries such as Ethiopia (Abate 1995; Tuno 2001). In the region studied, mushrooms were considered akin to tender meat due to their consistency but to have a better taste.

The Wixaritari favoured the mycorrhizal *Amanita basii* and *A. laurae* (species of the *A. caesarea* complex) followed by the lignicolous *Volvariella bombycina*, *Pleurotus djamor*, and *P. opuntiae*. Other fungi consumed included *Hypomyces lactifluorum*, an ascomycete that infects basidiomes of *Russulales*, and the phytopathogenic smut *Ustilago maydis*, a parasite of maize cobs and stalks. Terrestrial species such as *Agaricus campestris*, *Calvatia cyathiformis*, *Calvatia* sp., and *Marasmius oreades* were also important because they were the first to be consumed and are also found in large quantities. In addition, *M. oreades* is easy to dry under the sun and preserve for future consumption. Other ectomycorrhizal species such as *Butyriboletus frostii*, *Cantharellus cibarius*, *Lyophyllum* spp., *Ramaria* spp., *Rubroboletus dupainii*, and *Russula* spp. were also valued for their flavour, although they were not among the most liked mushrooms. *Amanita* sect. *Vaginata* (mycorrhizal), *Gymnopus* sp. (saprotrophic), and *Armillaria mellea* group (tree pathogen) were less commonly consumed and only collected if they were found along the road. Several species were not frequently eaten because of their consistency or flavour, including *Lentinus levis* which is chewy, and *Ganoderma oerstedii* which has a bitter flavour, although the latter was ground using a mealing stone (*metate*) and then combined with chilli to make a *pipián* dish (pumpkin seed sauce with toasted corn).

Mestizos preferred lignicolous species such as *Volvariella bombycina* and *P. djamor* but also consume some terrestrial ones such as *Agaricus campestris*, *Calvatia cyathiformis*, *Calvatia* sp., and *Marasmius oreades*. Few ate *P. opuntiae*, which, according to those interviewed, had a similar flavour to that of *P. djamor*. In addition, *Amanita basii*, *A. laurae*, *Cantharellus cibarius*, and *Ganoderma oerstedii* were eaten by mestizos who had been in close relationship with the Wixaritari or who, for different reasons, were located near a Wixarika community.

In Mexico, the species of the *Amanita caesarea* complex are among the most liked mushrooms, similar to the preferences of the Wixaritari (Burrola-Aguilar et al. 2012; Alonso-Aguilar et al. 2014; Quiñónez-Martínez et al. 2014). The mushrooms *Agaricus campestris*, *Calvatia cyathiformis*, and *M. oreades* are the most common terrestrial species traditionally consumed in Mexico, including Chihuahua (Moreno-Fuentes et al. 2004), Tlaxcala (Alonso-Aguilar et al. 2014; Montoya et al. 2003), and Oaxaca, where *M. oreades* mushrooms are also called *corralitos* (Jiménez et al. 2013). The frequency of consumption is most likely due to the species being found in pastures and compacted soils near urban areas (Osemwegie and Okhuoya 2011). Mushrooms were generally consumed among the entire population, beginning with the first appearances of *A. campestris* and *C. cyathiformis* basidiomes in June until September, after which *Volvariella bombycina* and *Boletales* species were found and consumed by the Wixaritari.

**Fig. 4:**
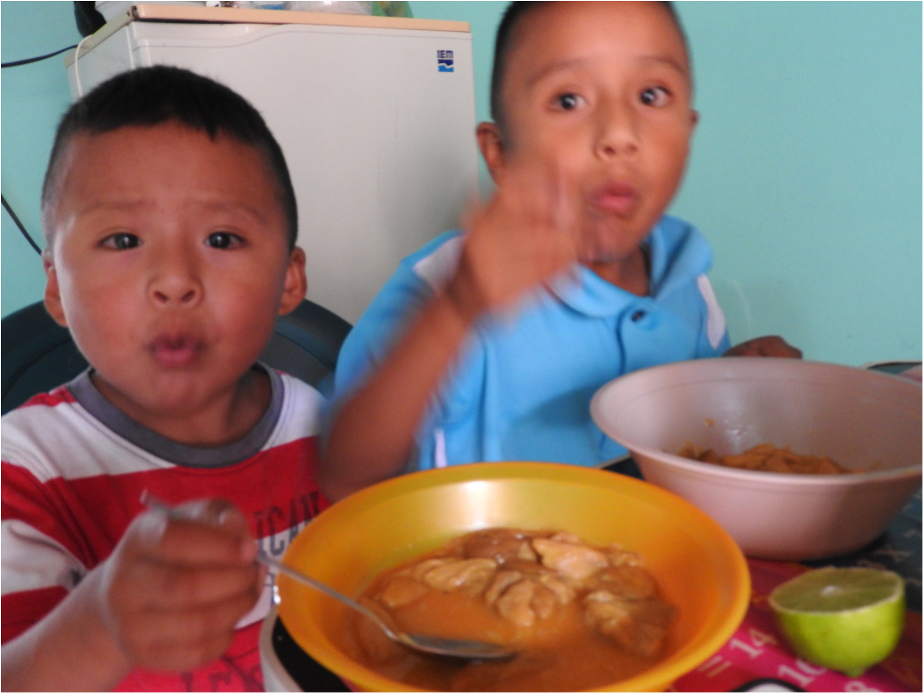
Saúl and Junior, Wixarika children eating *Amanita caesarea* complex with chilli

In isolated Wixaritari and mestizo communities, it was difficult to obtain a variety of foodstuffs, thus wild animals, mushrooms, and plants were important elements of the daily diet. As the municipality presented a high degree of marginalization (IIEG 2018), the preservation of the tradition of consuming wild mushrooms has enabled the local population to combat problems of hunger and malnutrition (Fig. 4). They represented an example of how wild edible mushrooms can be used to achieve food security in low-income areas (Boa 2005). Notably, mushrooms were not solely viewed as an emergency food, such as in countries of the Middle East during wartime or among those experiencing extreme poverty (Pierce and Emery 2005; Redzic et al. 2010), but rather as an exquisite food with excellent flavour.

Recipes form part of the cultural patrimony and the traditional knowledge transmitted orally through cultural practices that have endured for many decades (Sánchez-Martínez 2006). The mushroom recipes we encountered had been inherited and passed down for more than three generations by both women and men. In general, only one species was used per dish, and the modes of preparation were quite simple. They used elements within their reach, including different inexpensive ingredients typical of Mexican food such as tomatoes, onions, and chillis. The most elaborate dish was mushrooms with *mole* (a sauce prepared with many kinds of chillis) seasoned with wild plants such as *jocoyole* (*Oxalis corniculata*). However, simple recipes were preferred in which the flavour of the mushrooms stood out, so they were commonly prepared only with salt without any other seasoning or ingredient.

The Wixaritari consumed *Ustilago maydis* as a food or as ceremonial drink, commonly called *tsinari*. According to Torres (2000), *tsinari* is a drink (*atole*) made from the flour of white maize that is consumed for breakfast and, following Kindl (2003), it is a bitter *atole* for ritual use. In the present study, *tsinari* was found to be a maize drink containing *U. maydis*, known among the interviewees as *atole negro* (black *atole*), similar to the report of Villaseñor-Ibarra et al. (2018; as “*chinari*”). It was made from flour of nixtamalized maize —that goes through a chemical process in alkali solutions that increases its nutritional value— cooked with water in a clay pot over a fire until the mixture boiled and thickened. Before the mixture boiled, *U. maydis*, previously ground on a *metate*, was added to provide a drink with a black colour. This was stored in the clay pot in which it had been prepared, covered with a cloth, and placed in a cool and dark place, commonly in a hole in the ground lined with clay. In some variants of this recipe, unrefined brown sugar (*piloncillo*) was added to reduce the characteristic bitter taste.

Different conservation methods for mushrooms were documented. Mestizos often placed mushrooms in the refrigerator, although this only allowed them to be kept for 1 week approximately. Others prepared mushrooms in different dishes and froze them for consumption later, often until December when family members who had migrated to the USA returned home for the patron saint festivals. The Wixarika women conserved mushrooms by drying them under the sun; once dried, the mushrooms were stored in burlap sacks (*arpillas*). Similar reports of drying and conserving mushrooms are recorded among the Rarámuris, Otomíes, and communities in Tlaxcala, Nevado de Toluca, and Zacatlán, Puebla (Moreno-Fuentes 2014), as well as in other countries such as Nepal (Giri and Rana 2008) and Cameroon (Kinge et al. 2011).

The Mestizos and Wixaritari considered mushrooms to be rich in vitamins and nutrients and, therefore, to be a healthy food. This finding coincides with that of Bautista-González and Moreno-Fuentes (2014), who mention that edible mushrooms are considered medicinal or to be a medicinal food that prevents illnesses because of their high nutrient content. Like other wild foods, mushrooms were considered free of the chemical agents present in cultivated foods. This perception has been observed in central Mexico (Alonso-Aguilar et al. 2014; Robles-García et al. 2018), which stimulates the consumption of wild foods in a context of high use of agrochemicals as occurs in Mexico.

**Fig. 5:**
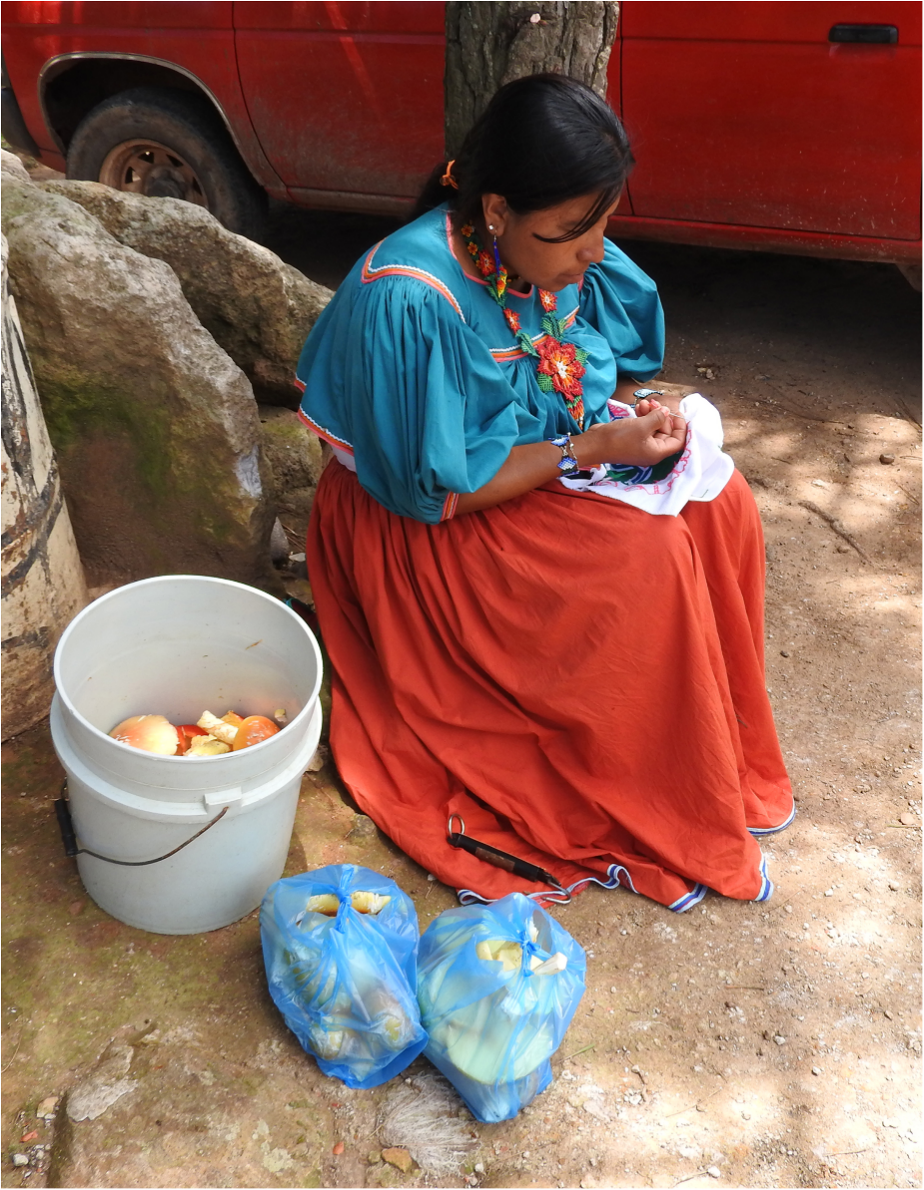
Wixarika woman selling *Amanita caesarea* complex

In contrast with the rural communities of central Mexico, where the sale of mushrooms is an important source of family income (Pérez-Moreno et al. 2008), the sale of mushrooms in the municipality was an insignificant source of extra income. The most frequently sold species were, in order of importance, *V. bombycina* and *P. djamor*, sold house-to-house mainly by mestizos in mountainous communities, including San Lorenzo de Atzqueltán and Izolta. These mushrooms were sold at high prices, compared with other natural products on sale, reaching $200 and $100 pesos (approx. 10 and 5 $US) per kg, respectively. *Amanita basii* and *A. laurae*, however, were sold at $70 and $50 pesos (approx. 3.5 and 2.5 $US) per kg, respectively, by the Wixaritari in neighbouring municipalities such as Mezquitic and Bolaños (Fig. 5). No other traditionally consumed species were seen being sold.

Several factors prevent the sale of mushrooms being a profitable activity despite their high prices. Most individuals prefer to collect their own or, on the other hand, access to collecting sites is difficult. Because of the effort collecting requires, most people prefer to consume them directly or to give them to family or close friends. There are consequently few specialists collecting mushrooms for sale, unlike in central Mexico where there are specialist called *hongueros* —mushroom experts, collectors, and sellers (Montoya et al. 2003, 2008; Ruan-Soto et al. 2006).

#### Ludic use

Child’s play may represent the first step in generating understanding and establishing a mycophilic relationship. Both Wixarika and mestizo children play with *Calvatia cyathiformis*, using them as balls or projectiles when collecting mushrooms with their parents or relatives. This practice has been the case for several generations: the elderly, adults, and young people sometimes playing together after a collecting trip before preparing the remaining mushrooms for the meal. Similar use of *Calvatia* and *Pisolithus*, like balls and projectiles, has been reported in Santa Catarina Estetla, Oaxaca (Hernández-Santiago et al. 2016).

#### Medicinal mushrooms

The mestizos do not use medicinal mushrooms, but the Wixaritari used two polypores and one bolete to alleviate particular conditions. The leathery *Ganoderma oerstedii* and *Pycnoporus sanguineus* are ground on a *metate*, mixed with water, and boiled in clay pots. The bolete, which we did not find and so could not be determined, is roasted on a *comal* (hotplate or griddle) and seasoned with salt for treating heart and joint problems. *Pycnoporus sanguineus* was used to treat skin conditions and fever; the liquid obtained from cooking this mushroom was applied to affected areas or used as a body wash in the case of fever.

A drink made from *Ganoderma oerstedii* was used as a remedy for stomach pain, intestinal diseases, and kidney problems. It could be drunk or applied with wet clothes on the stomach. Some species of *Ganoderma* have been used in traditional oriental medicine. It has been shown its high content of bioactive ingredients, as alkaloids, glycoproteins, sterols, triterpenoids, vitamins, among others, being effective in the treatment of a wide range of diseases (Paterson 2006).

Traditional mycological knowledge of medicinal mushrooms has been best conserved in the most marginalized, distant, and isolated communities. Although in our study area, national public health programmes encouraged individuals to live in larger communities with free access to medical services (IIEG 2018), some elderly Wixaritari remained reluctant to use modern medicines and continued to treat their diseases or afflictions with different plants and mushrooms.

#### Knowledge transmission

**Fig. 6:**
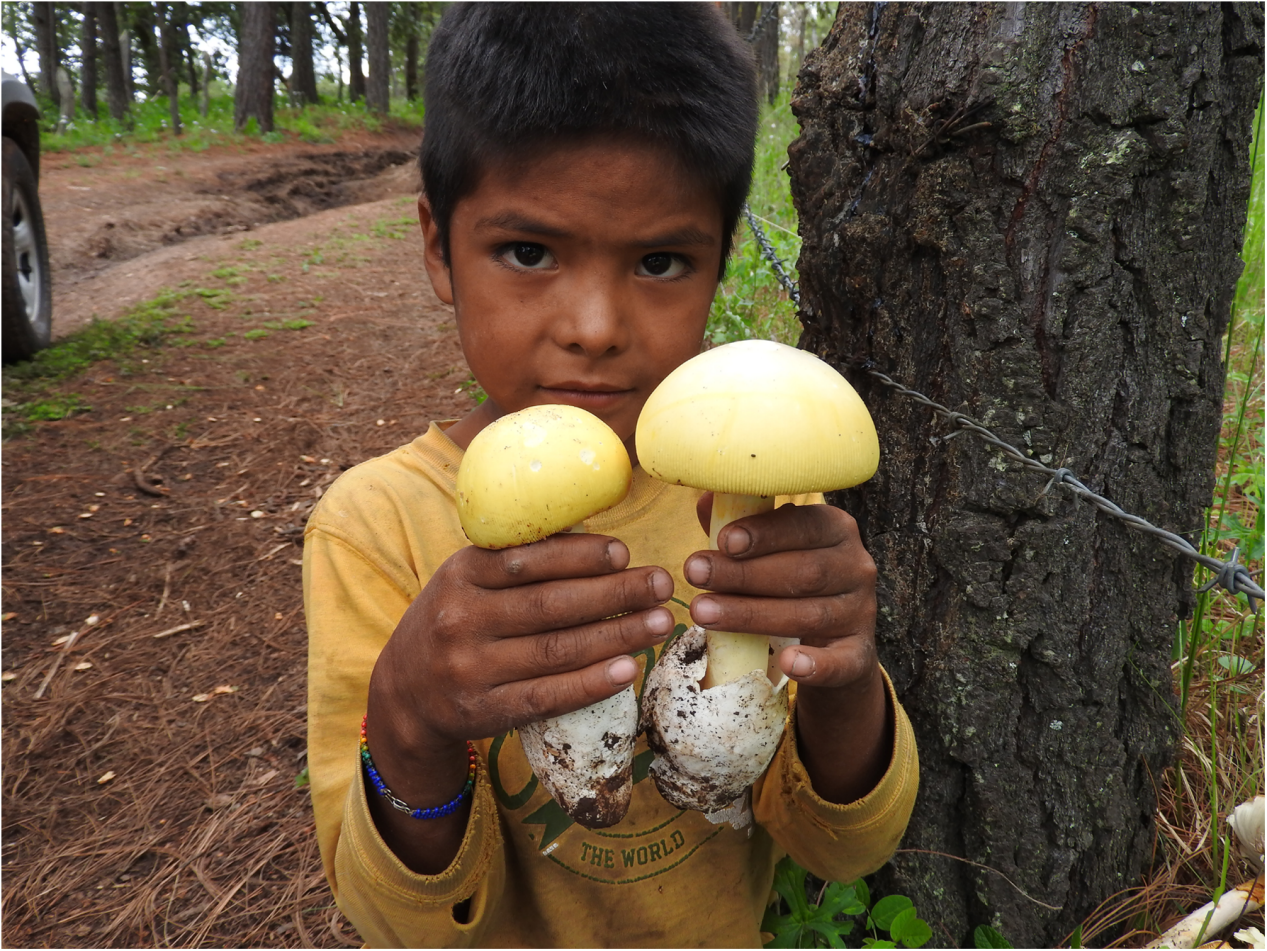
Alex, Wixarika child showing *Amanita laurae*

Parents or grandparents transmit traditional mycological knowledge to their children through participation. Children are taught to collect mushrooms in the company of adults, who show them the places and times where mushrooms are to be found and teach them which characteristics to observe to avoid harmful species (Fig. 6). Children are not prevented from touching mushrooms, even the ones that may be toxic. Rather, adults simply tell children which mushrooms cannot be eaten, and these are left in situ. Back home, they learn traditional recipes and preparation methods.

Adults valued being keepers and transmitters of traditional mycological knowledge and did not wish this to be lost within their families. However, they often felt that younger people did not share the same interest in learning about mushrooms as when they were young or that young people underestimated the value of wild resources. Traditional knowledge is being lost as result of economic changes, modernization, urbanization, and even formal education. We found these factors were leading to a cultural change and a lack of interest among the new generation in natural resources and traditional knowledge as already recognized elsewhere (Saynes-Vásquez et al. 2013).

#### Toxic mushrooms

The preservation of traditional mycological knowledge is fundamental to avoiding poisonings (Ruan-Soto et al. 2012). Mushroom poisonings or mycetisms were uncommon in this municipality because people only trusted mushrooms they know and transmitted the knowledge required to recognize edible versus toxic mushroom. Thus, they only trusted mushrooms that had been consumed for several generations. The municipal health centres did not have any official records of intoxication as a result of mushroom consumption.

The mestizos considered all coprophilous mushrooms and all species with black gills or scales to be toxic. The relationship of excrement with toxicity occurs in many communities, for instance in a tropical community in southern Mexico (Ruan-Soto et al. 2009). We observed that in communities settled in rocky soils without grasslands, terrestrial mushrooms were considered toxic, thus only lignicolous ones were consumed. In addition, a belief that no toxic mushrooms existed was also recorded; in that case, mushrooms were perceived to only cause illness in individuals who do not consume them regularly.

For the Wixaritari, a divine mushroom similar to each edible mushroom also exists. These mushrooms were considered God’s property, and people could become ill after consuming these species. When it was impossible to visually distinguish a divine mushroom from an edible one by macromorphology, they used smell. Similar practices are recorded in the temperate zones of central Mexico where an unpleasant smell and taste are associated with toxic mushrooms (Ramírez-Terrazo et al. 2014). On the other hand, some species considered toxic by the Wixaritari are consumed in other areas in Mexico. For example, *Cantharellus cinnabarinus* is traditionally consumed in Oaxaca (Garibay-Orijel et al. 2006) but is considered to be a *Hongo de Dios* (mushroom of God) or the *Dueño* (owner) of *C. cibarius* in Villa Guerrero.

Despite the lack of official records, testimonies of three individuals who suffered mycetism or heard of cases of intoxications with mushrooms were recorded, although the places and names of those affected were uncertain. Mestizos considered cultivated white button mushrooms to be toxic because of stories in which one woman died after consuming them and another woman from the locality of Santa Rita fell ill after consuming them in a restaurant. This latter woman also affirmed that, as a result of that event, she would no longer eat any kind of mushroom. This story had repercussions because the sale of white button mushrooms was then halted in the markets and stores of the municipality. Accordingly, and since then, it is not common to find this product and when on sale the locals do not buy it. Furthermore, it was believed among some families that domestic animals could take possession of the sickness and sacrifice themselves for their owners. In one reported case of intoxication, a mestizo child was considered to have only suffered minor complaints because his horse had died to save him.

The transmission of knowledge of how to recognize mushrooms had not been significantly modified in the region. The Wixaritari were able to identify clear distinctive characteristics of toxic versus edible mushrooms, mainly using visual clues and smell, but not the taste. This could explain why the intoxications reported by the Wixaritari were only caused by species of *Russula*. Species with red caps (pilei) which are traditionally eaten could be confused with the *R. emetica* group, which are similar at first sight but can be distinguished by their peppery taste (Hallock 2007). *Amanita* species with white basidiomes from the *A. bisporigera* group were seen in the study area but not collected as neither the Wixaritari nor the mestizos recognized them and did not give them any attention.

The treatment of intoxications, including the perception of the intoxication, differed between the cultural groups. Nevertheless, in both vomiting was seen as the remedy when symptoms presented; specific remedies to treat intoxications were not recorded.

#### Hierophanic mushrooms

We obtained a testimony of the use of psychoactive mushrooms by an elderly Wixarika woman as a hierophanic agent —one that gave a view of the divine. According to this woman and her family, the mushrooms selected her because their consumption by her was not premeditated. She received signs that drove her to eat a psychoactive species, and after ingestion began to experience manifestations such as voices and sounds that only she could perceive and considered sacred. Communication with the divine through the consumption of mushrooms has been reported in several parts of the world. One of the most familiar cases is the Mazatec shaman María Sabina who ate mushrooms to “see God” and performed healing ceremonies through ingesting mushrooms (Estrada 1989; Guzmán 2014). In contrast with the Mazatec people, the Wixaritari woman was able to maintain permanent communication with the divine being after eating mushrooms only once. This case accords with the Wixaritari credence in which gifts, including healing, are imparted at birth or acquired through a process of shamanic initiation (Neurath 2000), in this case by eating mushrooms.

The term of “entheogenic” has been applied to psychoactive mushrooms used as magical-religious agents (Ruck et al. 1979). Etymologically, entheogenic signifies “that which contains God or God within”. However, when the elderly woman ate the mushrooms, they were not viewed as necessarily containing God, but allowed her to establish communication with the divine beings. This is why we decided to use the term hierophanic, meaning “something sacred that is shown to us” (Eliade 1981). Hierophanies are manifestations of God or contact with God. This phenomenon is seen in other countries where neurotropic mushrooms are also used in rituals to establish a connection with sacred beings, yet the mushrooms do not represent or contain God. For example, in Siberia, *Amanita muscaria* is used to communicate with the souls of the dead and spirits in addition to treating illnesses, finding solutions to problems, and viewing the past, among other attributes (Saar 1991). In Oaxaca, the Chinantec people ingest species of *Psilocybe* to diagnose animals, locate objects, or to establish communication with a deceased loved one. The Mazatec and Chatino peoples say they can also see and speak with God after ingesting mushrooms (Guzmán 2016).

#### Wixarika conceptions of mushrooms

For the Wixaritari, mushrooms have divine connotations, autonomy, and feelings that make them similar to people. They consider mushrooms are able to breathe and to feel sadness, pain, or happiness, in addition to being able to see and perceive what occurs around them, which is expressed through changes in their physical state. For example, wilting is interpreted as a sign of sadness. According to Neurath (2000), *mara’akames* (shamans or spiritual leaders) can talk and communicate with all living things, and some people mentioned that mushrooms could speak, not with ordinary people but just with special ones such as *mara’akames*.

These characteristics are not peculiar to mushrooms but present in by all living things according to the Wixarika mythology (Neurath 2000). Villegas (2016) mention that, according to the Wixaritari, the different organisms and components of the universe can present human behaviours. Similarly, we found that the Wixaritari perceived mushrooms as beings analogous to people.

As noted above, mushrooms considered toxic were called *Hongos de Dios* (mushrooms from God) or *Dueños* (owners) in Spanish. In the Wixarika language, the epithets ‘*itaikarieya* or *kutsiyari* were added to the name of these mushrooms, inferring they are mythological beings or have spiritual qualities. While these species can be observed and touched, they do not belong to the earthly realm, but rather exist in an alternate and mythological space. These mushrooms establish communication with God, watching and protecting the site where they were found, and, importantly, are essential for the development of edible mushrooms species. This vision coincides with the previously reported Wixarika worldview wherein the world is understood as a whole, and the multiple facets of the world are connected with one another (Kindl 2003).

For the mestizos, the appearance of mushrooms is attributed to a combination of ecological factors that promote their growth and is always associated with rain. In contrast, the Wixaritari explain the origin within their myth of the origin of the universe in which animals, plants, and mushrooms all began to grow after a great rain. This view has previously been reported in ethnographic and anthropological works (Neurath 2002, 2016; Medina-Miranda 2017; Villegas 2016), and does not focus specifically on mushrooms.

There was a story known among the Wixaritari people older than 70 years, that God decides which mushrooms were suitable for human consumption; this arises from the idea that mushrooms are a divine gift and food that saves people from dying of hunger. This recalls the Lacandon myth in which God decides what can and cannot be eaten (Ruan-Soto et al. 2007). However, in the case of the Wixaritari, this idea formed part of a syncretic myth referring to the Catholic God (Neurath 2016).

## CONCLUSIONS

The Wixaritari and mestizos, two cultural groups in the same geographic region in contact with the same natural resources, presented differences in their conceptions and perceptions of mushrooms and in their use and understanding. These disparities reflected their distinct views of life. In both groups, mushrooms formed part of different social and cultural traditions and, for example, were eaten as food, used in games, and also formed part of different legends. Overall, mushrooms were valued and understood according to the worldview of each group.

The mestizo groups perceived and recognized the mushrooms nearby where they carried out their daily activities. The indigenous Wixaritari, in contrast, recognized and used a greater diversity of mushrooms whether they lived in forested areas or ones of disturbed natural vegetation. However, both groups had undergone cultural and social changes resulting in the loss of the knowledge and use of some mushrooms species.

Social modernization, especially the replacement of natural resources by cultivated ones, and modification of traditions had led to an interruption in the transmission of traditional knowledge. This process was also linked to the loss of a sense of belonging to certain natural ecosystems. We found evidence of changes in the level of traditional knowledge, implying changes to the degree of mycophilia. As a result, some individuals had stopped using and consuming wild mushrooms as they had become wary of them or were unable to recognize toxic ones. On the other hand, other individuals had adapted their traditional knowledge when migrating to new environments, although the use of some species not present in their new areas might be lost. Even so, in other cases, the importance of certain species was so great that, despite the scarcity or difficulty of finding them, individuals would find a way to buy or access them.

The two groups shared some aspects of mushroom use and knowledge as a result of cultural exchanges, co-existence, and shared historical events that had reduced previous barriers between them. Also, parallel knowledge was presented in many cases. For example, both groups were able to detail the collection, phenology, and ecology of the same species and also understood the role of fungi as degraders of organic matter and as essential components of nature and life-cycle. Both, consequently, understood the importance of conserving fungi species and their ecological roles. The traditional ecological knowledge of both cultural groups was fundamental to their use and collection practices. Knowing when and where to search for a particular species had enabled the harvest of mushrooms to become a family activity and, in some cases, a recreational one. The practices and customs surrounding mushroom picking have functioned as an effective means of transmitting knowledge concerning edible mushrooms and other fungi found during collection activities.

Although much work has been done to document traditional knowledge and uses of mushrooms in various parts of the world (e.g., Buyck and Nzigidahera 1995; Dugan 2011; Yamin-Pasternak 2011), more studies are needed to ensure much of potential value is not lost. Such knowledge is part of the identity of the peoples, and so the cataloguing uses, linguistics, myths, and harvesting can prevent their loss as modernization proceeds and in this case, by the dominance of a hegemonic culture. The preservation of this knowledge can promote a revaluation of wild mushrooms as resources and promote their conservation. Knowledge of useful wild species, such as edible ones, can also be revitalized and their use encouraged so making a greater contribution to food security, especially in marginalized regions.

## Data Availability

The datasets used and analysed during the current study are available from the corresponding author on reasonable request.

## Abbreviations

sect: section
SOLAE: Sociedad Latinoamericana de Etnobiología
sp.: species (singular)
spp.: species (plural)
